# Expl(AI)n It to Me – Explainable AI and Information Systems Research

**DOI:** 10.1007/s12599-021-00683-2

**Published:** 2021-02-16

**Authors:** Kevin Bauer, Oliver Hinz, Wil van der Aalst, Christof Weinhardt

**Affiliations:** 1 Leibniz Institute for Financial Research SAFE, Theodor-W.-Adorno-Platz 3, 60323 Frankfurt am Main, Germany; 2 Faculty of Economics and Business Administration, Goethe University Frankfurt, Theodor-W.-Adorno-Platz 4, 60323 Frankfurt am Main, Germany; 3 RWTH Aachen, Lehrstuhl für Informatik 9, Ahornstr. 55, 52056 Aachen, Germany; 4 Institute of Information and Systems and Marketing (IISM), Karlsruhe Institute of Technology (KIT), Kaiserstr. 89, 76131 Karlsruhe, Germany

## Introduction

The field of Artificial Intelligence has seen dramatic progress over the last 15 years. Using machine learning methods, software systems that automatically learn and improve relationships using digitized experience, researchers and practitioners alike have developed practical applications that are indispensable and strongly facilitate people's everyday life (Jordan and Mitchell 2015). Pervasive examples include object recognition (e.g., Facebook's Moments and Intel Security's True Key), natural language processing (e.g., DeepL and Google Translate), recommender systems (e.g., recommendations by Netflix or iTunes), and digital assistants (e.g., Alexa and Siri).

At their core, these applications have in common that highly complex and increasingly opaque networks of mathematical constructs are trained using historical data to make predictions about an uncertain state of the world. Based on large sets of labeled images, Deep Convolutional Neural Networks, for instance, can learn to make highly accurate individual-level predictions about the presence of diseases. This includes predicting positive COVID-19 patients (Shi et al. 2020). While highly accurate predictions in and of themselves are vital to informing fact-based decision-making (regarding disease detection even in a literal sense), the high predictive performance of state-of-the-art machine learning models generally comes at the expense of transparency and interpretability of their outputs (Voosen 2017; Du et al. 2019). Put differently: the majority of high-performance machine learning models are characterized by an incapability to convey human-interpretable information about how and why they produce specific predictions. Hence, such machine learning applications are often complete black boxes to their human users and even expert designers, who frequently lack an understanding of the reason behind decision-critical outputs.

From a methodological point of view, the inability to provide an explanation that accompanies specific predictions creates three types of high-level problems.

First, neglected opacity creates an immediate lack of accountability as it impedes the auditing of such systems' predictions. This shortcoming has sparked concerns about the rise of a black box society where opaque algorithmic decision-making processes in organizations and institutions entail unintended and unanticipated downstream ramifications, which change things for the worse (Pasquale 2015; Angwin et al. 2016; Obermeyer et al. 2019).

Second, the potential to enhance economic efficiency and human welfare using AI is not limited to informing specific decisions through predictions. Revealing new domain knowledge hidden in complex Big Data structures appears to be another extremely promising avenue (Teso and Hinz 2020). Hence, organizations and institutions may harness machine learning systems to confront human users with their own errors and teach them to improve their domain knowledge (Metcalfe 2017). To use machine learning applications to help humans widen their horizons of reasoning and understanding requires systems to explain their inherent reasoning in a human-understandable way that addresses the pitfalls of human learning processes.

Third, the black-box nature of machine learning applications can hamper their acceptance by users. This, in turn, likely impedes the integration of the application into existing processes. Naturally, reaping a technology's associated benefits presupposes its actual use that will not occur if systems' opacity inspires resistance and broad aversion. Especially in cases where the machine learning model's outputs contradict human experiences and intuitions, the provision of an interpretable explanation is of utmost importance to avert the emergence of tensions in human–machine collaboration and thereby resistance (Ribeiro et al. 2016).

Overcoming machine learning models' opacity and creating techniques that produce human-interpretable explanations whilst maintaining high predictive performance is not only a methodologically desirable objective. There are also immediate operational benefits from technological, social, economic, legal, and psychological perspectives. Specifically, model interpretability constitutes a binding constraint enabling (i) the optimization and debugging of models, (ii) the detection of inaccurate discriminatory patterns, (iii) the monitoring of continuous learning processes, (iv) the adoption of the technology by intended users, (v) accountability and responsibility, and (vi) users to harness models as teachers to enhance their knowledge and skills.

Considering that model interpretability is a key factor that will determine whether machine learning technologies can live up to their promise of unforeseen efficiency and welfare gains (Rahwan et al. 2019), it is not surprising that policymakers have caught on to this issue as well. With the General Data Protection Regulation (GDPR) that has taken effect in 2018, the European Union effectively provides people with a right to obtain an explanation about when and why an algorithm produced a specific, personally consequential decisions (Parliament and Council of the European Union 2016, Sect. 2, Art. 13–15, Sect. 4, Art. 21, 22; Goodman and Flaxman 2017). With the fast integration of ever-more complex machine learning applications into business processes, regulators will almost certainly introduce additional measures with which they intend to maintain legal oversight over algorithmic systems. As the (automatic) provision of human-readable explanations for algorithmic outputs arguably constitutes a natural angle to do so, the study and examination of interpretable machine learning using scientific tools are important from an operational compliance perspective as well.

## Interpretable Machine Learning

The examination and development of techniques that render the outputs of opaque, high performing machine learning models interpretable have gained increasing attention recently. A growing number of international conferences and workshops focus on sensitizing researchers and partitioners for the topic and combining complementary forces. Examples include IJCAI/ECAI Workshops on Explainable Artificial Intelligence, XCI on Explainable Computational Intelligence, ICAPS Workshop on EXplainable AI Planning and the Fairness, Accountability, and Transparency (FAT-ML) workshop. While researchers' and practitioners' attention for the field of interpretable machine learning, often more broadly referred to as Explainable Artificial Intelligence (Van Lent et al. 2004; Adadi and Berrada 2018), is steadily increasing, its origins can be traced back to the 1980s where there have already been efforts to explain outputs of Artificial Intelligence systems of the time (see Moore and Swartout 1988 for a survey). With the second AI-winter, however, such efforts largely ceased until rapid advancements over the last two decades have led to the integration of ever-more-powerful, but at the same time opaque, machine learning applications into almost every facet of people's everyday life. These novel methods have led to ethical, economic, and legal pressures associated with systems' opacity that inevitably renewed interest in the topic.

Today, the nascent research on interpretable machine learning broadly revolves around understanding the prerequisites and consequences of interpretability techniques that, in addition to allowing humans to observe specific outputs of opaque machine learning models, help to understand how these outcomes come to be. On the technical part, one can generally distinguish between research efforts involving intrinsic interpretability and post-hoc interpretability methods (Du et al. 2020). Research on intrinsic interpretability methods focuses on the development of models that are inherently self-explanatory and provide an immediate human-readable interpretation about how they transform certain inputs into outputs due to their structure. Logistic regressions and decision trees are examples of simple machine learning models that are intrinsically interpretable as humans can infer their inner logic from respectively examining regressor coefficients and logic classification conditions. Research on post-hoc interpretability methods, on the other hand, concerns itself with achieving the interpretability of a given complex machine learning model via the construction of a second surrogate model or method that approximates the behavior of the more complex model. Examples include LIME-based techniques (Ribeiro et al. 2016) and SHAP methodologies (Lundberg and Lee 2017) that rely on input perturbations to explain the model outputs. The main difference between intrinsic and post-hoc interpretability methods can mainly be found in the trade-off between prediction and explanation accuracy with the first potentially providing better explanations at the expense of predictive performance and vice versa for the latter. Notably, for some problems, it may also be the case that a combination of the two types of explanations is ideal.

Independent of whether an interpretability technique belongs to the class of intrinsically or post-hoc methods, the explanation can occur on a global or the individual level (Rodríguez-Pérez and Bajorath 2020). A global interpretation means that users can gain an understanding of a model's fundamental structure, underlying assumptions, and parameters that increases its overall transparency of working mechanisms. Local interpretability intends to illuminate the contribution of specific input features to the model output. This can contribute to identifying causal relationships in the data. Thereby users can better understand why a model makes a particular prediction.

Apart from technical aspects, there is a growing number of studies analyzing how to integrate interpretability techniques into decision-making processes and how such techniques interact with human users. So far, the majority of previous studies has primarily focused on how people respond to different types of explanations, subjectively measured intuitiveness and usability of specific interpretability methods, and whether the model interpretability can improve the performance of human decision making, see for example (Doshi-Velez and Kim 2017; Lage et al. 2019; Alufaisan et al. 2020; Shin 2021). The limited number of studies researching these questions indicates that interpretability techniques, to a varying degree depending on their representation and complexity, can improve people's perceived trustworthiness of machine learning models, their usability, and the optimality of their decisions. Research on the impact of model interpretability on human behavior and cognitive processes, such as learning, is extremely scarce. A notable exception is a study by Abel-Karim et al. (2020) that demonstrates how interpretable outputs by machine learning models can teach humans novel domain knowledge in the domain of medicine.

## Relevance for BISE Research

Advances in the field of interpretable machine learning are indispensable to enable machine learning applications to better serve humanity. Therefore, the increasing interest and recent developments in the field are extremely welcome as well as promising. Yet, interpretable machine learning as a field is still in its infancy and requires more scrutiny and rigorous scientific research. Many important questions remain and need to be addressed in the future. Especially when it comes to the interaction between interpretable machine learning and human learning of new domain knowledge, arguably one of machine learning applications' most promising and until recently mostly overlooked benefits for humankind, research is lagging behind.

The versatility of requirements and consequences that the presence (or absence) of model interpretability entails for individual decision-makers on a micro-level and the entire society on a macro-level, predestines Information Systems researchers to focus on the field of interpretable machine learning. This makes it a highly relevant and meaningful field for BISE research, especially when considering that the interest in understanding the working mechanisms of machine learning models steadily grows for both academic and industrial communities. Based on the outlined considerations, the different BISE departments can and have a responsibility to contribute to the advancement of interpretable machine learning so that machine learning technologies can live up to their promise of ultimately enhancing human well-being.

There are manifold and urgent avenues of future research for Information Systems researchers in the field of interpretable machine learning:

- **User-centric model interpretation:** As one of the central research foci of Information Systems researchers is the design of interactive, user-centric technologies and how they affect individuals, organizations, and societies at large, one natural direction is the advancement of current interpretability techniques to meet user demands. The majority of current designs meet their developers' demands but not their ultimate users' demands, who are typically domain, yet no technical experts. Here Information Systems researchers can make a valuable contribution by taking over a lead role in identifying and implementing the demands of different types of end-users.
- **Feedback effects from interpretability techniques:** Working at the intersection of sociology, economics, psychology, and computer science, Information Systems researchers are particularly suited to study how the disclosure of machine learning application's inner workings to users may influence their behaviors in domains similar, however, not identical to the one where the machine augments their decision-making. It is crucial to understand whether, and if so how, interpretability techniques may fundamentally change users' beliefs and preference structure, thereby possibly creating unanticipated spillover effects with significant downstream consequences.
- **A Lucas' critique:** Along the lines of an argument by the Nobel laureate Robert Lucas from the 1970s, acting upon or immediately revealing insights about the functioning of a system will likely cause the system's functioning to change and thereby render previous insights mute. The European Union's General Data Protection Regulation already stipulates that algorithmic systems' targets have a right to information. If the disclosure of high-performing machine learning models' inner workings by means of interpretability techniques to targets entail such consequences, the broad adoption of interpretable machine learning methods may create endogenous concept drifts. Examining the existence of such side-effects of model interpretability and how to mitigate them constitutes a fruitful avenue for future research.

This list is by no means exhaustive and only represents a fraction of research directions that Information Systems researchers may adopt. Yet, it emphasizes the important role that BISE research can play.
